# Guidelines of Guidelines: Conservative, Pharmacological, and Surgical Management for Neurogenic Lower Urinary Tract Dysfunction

**DOI:** 10.5152/tud.2024.23232

**Published:** 2024-03-01

**Authors:** Diana Carolina Ochoa, Alessia Christina Mahoney, Christina Fontaine, Hashim Hashim

**Affiliations:** 1 Department of Bristol Urological Institute, Southmead Hospital, Bristol, UK; 2 Cardiff University School of Medicine, Cardiff, UK; 3 Southmead Hospital, Bristol, UK

**Keywords:** Neurourology, surgical treatment, conservative treatment, pharmacological treatment

## Abstract

Neurogenic lower urinary tract dysfunction (NLUTD) encompasses a broad spectrum of neurological conditions affecting the lower urinary tract. Managing NLUTD requires a tailored approach focused on preserving kidney function and enhancing patients’ quality of life. Clinical guidelines provide valuable guidance for healthcare professionals, but discrepancies in recommendations arise among other factors due to limited high-quality clinical evidence. Prominent guidelines from organisations like the International Consultation of Incontinence, the European Association of Urology, the American Urological Association, and the National Institute for Health and Care Excellence offer varying recommendations for NLUTD management. This study reviews and summarizes the recommendations for conservative, pharmacological, and surgical management options across these guidelines.

## Introduction

Neurogenic lower urinary tract dysfunction (NLUTD) encompasses a wide range of neurological conditions that impact the lower urinary tract. This diverse population experiences variations in causes, pathology onset, bladder dysfunction, and progression likelihood.

Managing NLUTD requires a tailored stepwise approach with a focus on preserving kidney function and addressing symptoms like incontinence, voiding dysfunction, and urinary tract infections while prioritizing the patients’ quality of life, which is paramount in this group.

Clinical guidelines are grounded in the highest quality of available evidence. While a range of evidence-based treatment options for NLUTD management exists, the evidence is predominantly derived from observational studies and clinical investigations with weak methodological designs. Prominent guidelines in this domain are established by the International Consultation of Incontinence (ICI), the European Association of Urology (EAU), the American Urological Association (AUA), and the National Institute for Health and Care Excellence (NICE).

Discrepancies in recommendations arise due to different factors; scarcity of high-quality clinical evidence as well as the influence by the socioeconomics of the regions in which they are developed, and the nature of the health-care system, i.e., public vs. private vs. mixed models. Such discrepancies can create uncertainty for healthcare professionals and patients and hinder the standardization of care. To address this challenge, we assessed the concordance of prominent guidelines, aiming to elucidate the similarities and disparities in their treatment recommendations.

## Methods

A thorough review of the conservative, pharmacological, and surgical management recommendations on NLUTD from the most prominent guidelines was undertaken. The most recent update of the seventh ICI (2021), EAU (2022), AUA (2021), and the NICE 2019 was reviewed and summarized.

### Guidelines Recommendations

Behavioral Interventions are listed in Table 1.

Assisted bladder emptying: EAU and ICI guidelines contemplate Crede, Valsalva, and triggered reflex voiding (Table 1). However, EAU states that it would be considered only if urodynamically safe and warns of the risk of inducing autonomic dysreflexia and worsening pelvic floor weakness.^1^ Alongside treatment, EAU encourages patient education and surveillance techniques.^2^

Pelvic floor muscle training: All guidelines agree on its use and suggest combining treatment with electromyographic biofeedback and/or electrostimulation, as dual therapy has been proven to be more effective.^3^ National Institute for Health and Care Excellence only advocates this technique in SCI (spinal cord injury) or MS, and the EAU recommend it only in MS patients.

Catheters and appliances are listed in Table 2.

Intermittent Self-Catheterization: EAU and ICI guidelines favor the use of ISC compared to indwelling catheters (IDCs) but differ in their choice of catheter and its indications. European Association of Urology highlights that aseptic intermittent self-catheterization (ISC) reduces urinary tract infections (UTI).^4^ American Urological Association also favors the use of ISC compared to IDC but stresses the risk of bias. It also mentions that ISC has been associated with worse quality of life than IDCs—observed in the SCI population.^5^ Across all guidelines, the importance of counseling the patients for ISC before botulinum toxin therapy is highlighted.

Indwelling catheters: All the guidelines suggest avoiding indwelling transurethral and suprapubic catheterization (SPC). However, ICI agrees to use it to an extent. If it is necessary, AUA makes a strong recommendation of SPC over an indwelling catheter. European Association of Urology stresses the increased chance of UTIs. International Consultation of Incontinence and AUA make recommendations against antibiotic prophylaxis.

Other considerations about catheter valve, urethral plugs, condom catheters, pads, and occlusive devices are described in the different guidelines (Table 2).

Pharmacotherapy guidelines are detailed in Table 3.

Anticholinergics: indicated across the four guidelines for patients with neurogenic detrusor overactivity (NDO). The EAU suggests employing antimuscarinics in combinations to maximize outcomes and advocates using oxybutynin, trospium, tolterodine, and propiverine due to their efficacy and long-term tolerability.^5^ Alongside these, in patients with SCI and MS experiencing NDO, Darifenacin, and Solifenacin^6^ have been found to respond similarly. National Institute for Health and Care Excellence makes a weaker recommendation for their use in progressive brain conditions and draws on three main warnings: cognitive impairment, UTIs as reducing bladder emptying, and constipation. American Urological Association recommends antimuscarinics alone, or in combination with beta-3 adrenergic receptor agonists. It also recognizes the potential risk of cognitive impairment/dementia with its long-term use, therefore recommends shared patient decision making, as well as switching to other agents that do not cross the blood–brain barrier or combination of anticholinergics with alpha-blockers.^7^ Additionally, EAU adds alternative modes of administration as intravesical to minimize side effects.^8^

B3 agonists:recommended in isolation or combined with anticholinergics. However, EAU, ICI, and AUA mention conditional recommendation as monotherapy has shown inconclusive results.^9^

Cannabinoids: mentioned in EAU as an option. On ICI, the grade of recommendation is C.

Cholinergics: As mentioned by the EAU, bethanechol and distigmine enhance detrusor contractility and promote bladder emptying^10^ and is neither is frequently used in clinical practice. The level of evidence for their use is 2b.

Alpha blockers:All 4 guidelines hold a slightly different stance (Table 3).

Duloxetine: not mentioned in any of the four guidelines.

Minimally invasive treatment options are listed in Table 4.

Electrostimulation: EAU mentions transcutaneous electrical nerve stimulation, dorsal genital nerve stimulation, transcranial magnetic stimulation, pudendal nerve electrical stimulation, interferential medium frequency current electrical stimulation, and neuromuscular electrical stimulation but they reserve their full support as there are limited reports proving efficacy.

Intravesical electrostimulation: EAU and ICI agree on their indication to improve the voiding phase^11^ (Table 4). Unlike ICI, EAU also advocates for its application in patients with incomplete SCI or myelomeningocele.

Intravesical therapy:Recommended by EAU as an alternative route for antimuscarinics. International Consultation of Incontinence and NICE do not provide graded recommendations for alternative forms of antimuscarinic administration. Vanilloids and capsaicin have no current indication as their safety is not favorable.^12^Cannabinoids—only preclinical studies have detected its benefits.^13^

## Botulinum Toxin Injections

*Bladder:* All four guidelines broadly agree on intradetrusor botulinum toxin injections for NDO. European Association of Urology, AUA, and NICE advocate its use in SCI and MS. In contrast, the ICI recommends it independent of the underlying neurological condition. To maximize effectiveness, EAU suggests alternating between brands (Botox® to Dysport®) in case of failure.^13^ As grade A evidence, AUA suggests continued efficacy with repeat injections. Due to weaker evidence in other neurologic conditions, AUA suggests it may be offered, for example in PD, CVA, and spina bifida, as well as those with persistent NDO after augmentation enterocystoplasty.

Pelvic floor: The guidelines do not mention pelvic floor injections for NLUTD.

Surgical management is detailed in Table 5.

## Stress Urinary Incontinence

Before treatment selection, several factors should be considered, such as the severity of stress urinary incontinence (SUI), neurological impairment, possibility of progression, hand function, and ability to self-catheterize that needs to be present before the surgery according to EAU and ICI. At the same time, AUA suggests that only patients who can void on their own should be considered for sling surgery.

Urodynamics is strongly recommended. American Urological Association states that candidates should have acceptable storage parameters, and both ICI and AUA suggest using an occlusion catheter during UDS if necessary. European Association of Urology, AUA, and ICI report a higher incidence of de novo urgency in the neurogenic population after sling placement.

## Urethral Slings

Synthetic slings:In female patients, NICE does not recommend its use due to the risk of urethral erosion. In contrast, the EAU considers retropubic and transobturator tapes as alternatives in selected patients. The AUA suggests it should be avoided if there is a concern for future ISC and does not recommend its use as an occlusive sling. Optional for male patients, according to EAU.

Autologous slings:Procedure of choice in neurogenic female patients for EAU and NICE. They are stated as an option for males in EAU. International Consultation of Incontinence mentions that it is a preferable option with the same evidence of synthetic slings, and AUA suggests it should be used where an occlusive sling is considered.

## Artificial Urinary Sphincter

The most common procedure in male SUI patients, with a high success rate.^14^ The complication rate is higher than in the non-neurogenic population.^15^ Therefore, adequate counseling/discussion with the patient is stressed across the guidelines.

European Association of Urology and ICI strongly recommend its use in male patients with neurogenic SUI. According to AUA, it is an option for selected patients (male and female). National Institute for Health and Care Excellence recommends its use only if an alternative procedure, such as an autologous fascial sling, is less likely to control incontinence (gender not mentioned). It recommends monitoring the upper tract with annual ultrasound on follow-up. American Urological Association stresses the risks of erosion with ISC, which may be reduced by bladder neck cuff location.

The laparoscopic and robot-assisted approach is promising and has increased its use in the female neurogenic population. Long-term surgical and patient-reported outcomes are still needed. European Association of Urology supports its use in selected female patients in experienced centers. American Urological Association states that a robotic approach is an attractive option. A transvaginal cuff is considered a poor option due to the high risk of infection.

## Adjustable Continence Mechanisms (Pro-ACT /ACT)

In neurogenic population, it is considered experimental by AUA, and EAU mentions a lower cure rate with a higher complication rate when compared to non-neurogenic patients. Similarly, ICI states limited experience in neurogenic population.

## Bulking

American Urological Association and ICI agreed that it showed modest efficacy and poor long-term outcomes. American Urological Association stresses the low-evidence studies in male neurogenic populations. European Association of Urology advocates early positive results with an early loss of continence in both females and male. Also, AUA emphasizes that it is unclear how ISC would impact the outcome.

## Bladder Neck Closure

According to the ICI guidelines, it is performed mainly in children. It should be offered along with SPC, continent catheterizable stoma, or urinary diversion. It is considered an alternative in AUA for refractory cases or severe urethral pathologies. American Urological Association also stresses the importance of discussing the need for assisted reproduction.

## Bladder Outlet Obstruction

### Transurethral Resection of the Prostate

Careful selection is recommended across the guidelines. According to ICI and EAU, bladder outlet obstruction must be proven, and sphincter function assessed due to the high risk of de novo or persistent incontinence. International Consultation of Incontinence also states that transurethral resection of the prostate is an option in patients with Parkinson’s and mentions the lack of evidence to support the indication in patients with cerebrovascular diseases. Additionally, multiple system atrophy must be excluded due to the high risk of stress incontinence.

### Bladder Neck Resection

It is recommended by EAU only if a sclerotic ring in the bladder neck is identified along with proven functional obstruction. Also, ICI states that there is a lack of evidence. It is not mentioned in AUA or NICE guidelines.

### Urethrotomy

For urethral strictures, the treatment will be similar to the non-neurogenic population. A tailored stepwise approach is recommended, always considering the higher risk of needing intermittent catheterization. European Association of Urology recommends cold knife or neodymium/Yttrium Aluminum Garnet (YAG) contact laser at twelve o’clock. Urethroplasty should be considered in recurrent cases, according to ICI and EAU.

### Sphincterotomy

It is recommended across the guidelines in appropriately selected male patients. It increases the effectiveness of bladder emptying, reducing UTI’s, autonomic dysreflexia and vesicoureteral reflux.^16^ International Consultation of Incontinence stresses that the decrease of intravesical pressure is often unsatisfactory. According to AUA, ICI, and EAU, regular follow-up is required due to the risk of recurrence and additional treatment.^17^

American Urological Association and EAU recommend it at the 12 o’clock position with electrocautery resection or neodymium, YAG laser incision in patients who experience reflex voiding and can maintain a condom catheter, have poor hand function, or are unwilling to perform ISC.

### Bladder Neck Incision

It is contemplated by the EAU guidelines only for fibrosis at the bladder-neck level. It is not recommended in patients with detrusor hypertrophy as it causes thickening of the bladder neck.

### Botulinum Toxin into the Urethral Sphincter

Considered for the treatment of detrusor sphincter dyssynergia by EAU and ICI as the efficacy reported is high with few adverse effects.^18^ However, it is stressed that its effect is temporary, the optimal dose and mode of injection are still unclear, and it is not licensed for this purpose. American Urological Association considers chemical sphincterotomy to have limited efficacy over time, and therefore, it is not recommended for routine management of DSD in NLUTD.

### Urethral Stents

According to EAU, its effect is comparable with sphincterotomy with a shorter duration of surgery and hospital stay, and the continence relies on the bladder neck. International Consultation of Incontinence and EAU describe limiting factors such as costs, complications, and the need for reintervention.

### Balloon Urethral Dilation

European Association of Urology declared no further reports since 1994; therefore, it is no longer recommended.

## Denervation, Deafferentation, and Neuromodulation

### Sacral Anterior Root Stimulation

Produces detrusor and sphincter contraction; the latter relaxes faster, and “post-stimulus voiding” occurs.^19^ It requires a sacral deafferentation (dorsal rhizotomy) to control detrusor overactivity. It can also induce defecation or erection. Charcot spinal arthropathy is a potential long-term complication.^20^ According to EAU and ICI, candidates are patients with complete spinal cord injury above the implant location. American Urological Association states that it has promising outcomes. However, it is limited to investigational settings or specialty centers.

### Percutaneous Tibial Nerve Evaluation

European Association of Urology, AUA, and ICI guidelines advocate its use but differ in their choice of delivery method 27-30). American Urological Association recommend its use in NLUTD patients with storage symptoms and spontaneous voiding, as it has shown benefits in MS,^21^ PD,^22^ and CVA^23^ with isolated storage symptoms. It is currently approved for non-neurogenic OAB.

### Sacral Neuromodulation

According to EAU and ICI, there is growing evidence, but it is still unclear which patients are most suitable. National Institute for Health and Care Excellence mentions it may be used in patients for whom conservative treatments have been unsuccessful. American Urological Association states that sacral neuromodulation (SNM) is not recommended for patients with complete spinal cord injury or spina bifida. However, it may be considered for storage symptoms in MS, CVA, and PD. There is also limited evidence for its use in other mixed neurologic diseases including cerebral palsy, acquired brain injuries, viral and vascular myelitis, encephalitis, central nervous system tumor, incomplete spinal cord injury, multisystem atrophy, and spinocerebellar atrophy. For voiding dysfunction, treatment may be considered in MS. Additionally, there must be consideration that in progressive disease, such as with MS, there may be concurrent worsening of NLUTD and loss of efficacy.^24^

### Major Surgery

#### Detrusor Myectomy/Auto-augmentation

As mentioned by EAU and ICI, it reduces detrusor overactivity and improves compliance. It was mainly used historically and in the pediatric population.

#### Bladder Augmentation

It is recommended across the 4 guidelines in patients refractory to less invasive therapies for detrusor overactivity and/or poor bladder compliance and small bladder capacity. National Institute for Health and Care Excellence specifies that its indication is for non-progressive neurological disorders.

It is related to quality-of-life improvement and long-term renal function stabilization.^25^ Hand and cognitive function are essential as ISC is mandatory. It also requires lifelong follow-up due to its potential and long-term risks (bowel dysfunction (15%), mucus production (12.5%), stones (10%), metabolic abnormalities (3.35%), and bladder perforation (1.9%)). The risk of bladder cancer is 0.6%-4.5%.^26^ American Urological Association does not recommend surveillance cystoscopy in asymptomatic patients. European Association of Urology and ICI recommend special attention to patients with preoperative renal scars at higher risk of metabolic acidosis.

European Association of Urology recommends a prior supra-trigonal cystectomy in patients with a severely thick and fibrotic bladder wall, then a substitution cystoplasty. International Consultation of Incontinence, AUA, or NICE do not mention specific recommendations.

#### Continent Catheterizable Channels

European Association of Urology and ICI state it should be the first choice for urinary diversion in patients with limited dexterity, devastated urethra, or preference. American Urological Association mentions it is an option to select NLUTD patients with or without augmentation. European Association of Urology and ICI also emphasize complications and the high rate of reoperation rate.

#### Cystectomy

American Urological Association strongly recommends concurrent supra trigonal cystectomy or cystoprostatectomy at the time of urinary diversion in male NLUTD, as the delayed reoperation rate could be up to 50% due to empyema. The “Spence procedure” (vesicovaginal fistula) allows for drainage in female patients. European Association of Urology and NICE mentioned it as advisable to avoid pyocystitis.

#### Incontinent Urinary Diversion

Considered by EAU, ICI, and NICE in patients who are wheelchair-bound or bed-ridden with untreatable incontinence, upper tract severely compromised with impaired renal function, lower urinary tract destruction, unable to catheterize, and in patients who refuse other therapy. American Urological Association considers it as a last resort in those patients where there has been a failure to provide safe and adequate storage function. Due to the high risk of long-term complications, these patients’ continued long-term follow-up is imperative.

#### Ileovesicostomy

Considered by AUA for NLUTD patients unable to ISC. Counseling is essential due to the high risk of additional treatment or surgery. It is a reversible procedure. However, its drainage may be poor, with an increased risk of stones.

#### Undiversion

European Association of Urology considers that long-standing diversions may be successfully undiverted or an incontinent diversion changed to a continent one. However, ICI states that it is rarely indicated, requires meticulous planning, and has no evidence to date.

#### Other Surgical Procedures

European Association of Urology describes a series of procedures to restore continence and function, with some evidence of their use in the neurogenic population: 1) functional autologous sphincter using gracilis muscle and electrical stimulation^27^ restoring control over the urethral closure; 2) bladder neck and urethra reconstruction; Young–Dees–Leadbetter and Kropp urethra lengthening; 3) covering the bladder with striated muscle rectus abdominis, latissimus dorsi. International Consultation of Incontinence considers bladder neck reconstruction mainly in the pediatric population combined with SPC or urinary diversion. However, due to insufficient evidence in neurogenic patients, ICI does not recommend latissimus dorsi/rectus abdominis. AUA states these procedures are investigational due to their infancy in development or lack of adequate data and suggests that it should only be performed in a well-designed clinical trial.

## Conclusion

While guidelines agree broadly on standardized treatments, discrepancies emerge due to the scarcity of high-quality clinical evidence and the wide spectrum of neurogenic diseases included in a single guideline. Moreover, practices in different countries vary as well as the processes of production of the guidelines. Urgent research efforts are needed to strengthen the evidence base for NLUTD and improve patient care and outcomes. This will facilitate the development of more consistent and robust treatment recommendations across guidelines, ultimately benefiting NLUTD patients facing diverse challenges.

## Figures and Tables

**Table 1.** Behavioral Interventions

	NICE	EAU	ICI	AUA
Triggered reflex voiding	N/A	N/A	Grade of recommendation: C. Consider in patients with UI due to DO, without DSD	N/A
Behavioral treatment	Consider Bladder Retraining, timed voiding and habit retraining Suggest only after assessment by a NLUTD-trained professional alongside with education to family and carers. Particularly to those with cognitive impairment.	Only to patients with Parkinson’s disease.	Initial treatment SUI or DOI with negligible PVR and no DSD Grade of recommendation: C.	N/A
Pelvic floor muscle training	Consider in patients whose neurological conditions have preserved the ability to voluntarily contract the pelvic floor. Patients must undertake a specialist pelvic floor assessment. Combine treatment with EMG biofeedback and/or electrostimulation of the pelvic floor.	Combine treatment with EMG biofeedback and/or peripheral temporary electrostimulation	N/A	Recommend to selected patients, particularly MS or CVA. Conditional recommendation Evidence level: grade C.

AUA, American Urological Association; CVA, Cerebrovascular Accident; DO, Detrusor overactivity; DOI, Detrusor Overactivity incontinence; DSD, Detrusor Sphincter dyssynergia; EAU, European Association of Urology; EMG, electromyographic; ICI, International Consultation of Incontinence; MS, Multiple sclerosis; NICE, National Institute for Health and Care Excellence; NLUTD, neurogenic lower urinary tract dysfunction; PVR, Post-void Residual; UI, urinary incontinence; SUI, stress urinary incontinence.

**Table 2.** Catheters and Appliances

	NICE	EAU	ICI	AUA
Intermittent self-catheterization	Favored compared to indwelling catheters	N/A	Alongside antimuscarinics for patients with UI due to DO with no DSD. UI associated with poor bladder emptying (significant PVR)—Grade of recommendation: A.	Strong recommendation compared to ID catheters. (strong recommendation; evidence level: grade C) Aseptic technique. Daily prophylactic antibiotics are only recommended for patient with rUTIs (evidence level: grade C). Offer bladder instillations if recurrent UTIs. Initiate oral antimicrobial prophylaxis if rUTIs after discussing risk of antibiotic resistance.(conditional recommendation; evidence level: grade C) Potentially required with botulinum toxin therapy.
Indwelling catheters	Avoid transurethral or suprapubic. Antibiotic prophylaxis should not be offered routinely. Considered if symptomatic UTI after catheter change	Recommend a silicone catheter if used.	May still be given if DOI (with negligible PVR), combined with AM	Suprapubic catheterization over an indwelling urethral catheter if necessary—strong recommendation, evidence level: grade C. Do not to perform screening/surveillance cystoscopy (grade B). Imaging every 1-2 years or if susceptibility to calculi. Antibiotic prophylaxis should not be offered routinely.
Catheter valve	Alternative to a drainage bag.	Urethral plugs or valves has not been used in NLUTD patients.	N/A	Implanted catheters continue to be studied. Intraurethral valve pump device does not have an indication for NLUTD as of yet.
Condom catheters	N/A	Consider condom catheters.	N/A	
Pads	Consider absorbent pads		Suggest for DOI with negligible PVR, depending on co-operation and mobility—grade of recommendation B. Suggest for patients with suprapontine pathology if symptoms remain after behavioral therapy and medication	Can be used for a pad test at initial evaluation in patients with NLUTD.
Penile clamps	N/A	Do not give penile clamps to patients with NDO or low bladder compliance.	N/A	N/A

AUA, American Urological Association; EAU, European Association of Urology; ICI, International Consultation of Incontinence; NDO, neurogenic detrusor overactivity; NICE, National Institute for Health and Care Excellence; NLUTD, neurogenic lower urinary tract dysfunction; UI, urinary incontinence; UTI, urinary tract infection.

**Table 3.** Pharmacotherapy

	NICE	EAU	ICI	AUA
Anticholinergics	SCI and suprapontine conditions and symptoms of OAB. Urodynamic detrusor overactivity Monitor residual urine volume	Oxybutynin, trospium, tolterodine, and propiverine. Darifenacin and solifenacin in SCI and MS “Strong recommendation.”	First line for NDO. Grade of recommendation: A.	Consider as first line for NDO. Recommended alone or combined with beta-3 adrenergic receptor agonists Conditional recommendation; evidence level: grade C
B3 Agonists	N/A	Suggested in patients with NLUTD in isolation or combined with anticholinergics. Monotherapy has shown unclear results.	Prescribe for patients with suprapontine lesion DOI and negligible PVR.	Conditional recommendation with an evidence GR C
Cannabinoids	N/A	Considered for specialist management in patients with UI.	Grade of Recommendation of C.	N/A
Cholinergics	N/A	Bethanechol and distigmine not frequently used in clinical practice.	N/A	N/A
Alpha blocker	Do not prescribe for patients with bladder emptying problems as a consequence of neurological disease.	Strong recommendation	Consider in patients where catheters, behavioral modification, external appliances, do not work—grade of recommendation: C.	Consider to improve voiding parameters—conditional recommendation with an evidence level of C.

AUA, American Urological Association; EAU, European Association of Urology; ICI, International Consultation of Incontinence; NDO, neurogenic detrusor overactivity; NICE, National Institute for Health and Care Excellence; NLUTD, neurogenic lower urinary tract dysfunction; OAB, Overactive Bladder.

**Table 4.** Minimally Invasive Treatment

	NICE	EAU	ICI	AUA
Transcutaneous electrical nerve stimulation	N/A	Recognizes the benefits in NLUTD— more randomized controlled trials needed.	N/A	N/A
Dorsal genital nerve stimulation	N/A	Not fully supported at present. Based on an SR for SCI higher bladder capacities and inhibition of DO	N/A	N/A
Intravesical electrostimulation	N/A	Considered in detrusor underactivity or sphincter overactivity.	N/A	N/A
Transcranial magnetic stimulation	N/A	Under investigation.	N/A	N/A
Pudendal nerve electrical stimulation	N/A	Consider PNES.	N/A	N/A
Interferential medium frequency current electrical stimulation	N/A	Consider IMFC ES.	N/A	N/A
Neuromuscular electrical stimulation	N/A	MS is best coupled with PFMT and EMG biofeedback, compared to single therapy. Intravaginal electrostimulation and PFMT dual therapy is no more effective compared to single PFMT.	N/A	N/A
Intravesical antimuscarinics	N/A	Can be administered intravesically to reduce DO. Its efficacy, safety, and tolerability improved as a result of the different method of metabolism.	N/A	N/A
Vanilloids	N/A	No current indication for vanilloids.	N/A	N/A
Capsaicin	N/A	No current indication for capsaicin.	N/A	N/A
Resiniferatoxin	N/A	No for intravesical therapy.	N/A	N/A
Cannabinoids	N/A	Only beneficial in preclinical studies.	N/A	N/A
Bladder botulinum toxin injections	Recommended for NLUTD patients with spinal cord injury or multiple sclerosis refractory to oral medications. Consider if medical treatments fail or are poorly tolerated. Consider its use in children and young people. Residual urine volume should be monitored in those not catheterized during treatment. The upper urinary tract should be observed in those at risk of renal complications.	Recommended in SCI and MS. Carry out urodynamic studies in patients with maximal filling pressure of >40 cm H_2_O to monitor the effect of the injections on bladder pressure. Alternate between toxin brands	To be suggested. Non-invasive (catheters, behavioral modification, external appliances) techniques must also be found not to work.	Offer to patients with SCI and MS. Strong recommendation; grade A. Conditional recommendation for other neurological conditions with grade C.

AUA, American Urological Association; EAU, European Association of Urology; ICI, International Consultation of Incontinence; IMC ES, Interferential Medium Frequency Current Electrical Stimulation; NDO, neurogenic detrusor overactivity; NICE, National Institute for Health and Care Excellence; NLUTD, neurogenic lower urinary tract dysfunction; PMMT, Pelvic Floor muscle training; PNES, Pudendal nerve electrical stimulation; SR, Systematic review.

**Table 5.** Surgical Treatment

Surgical Treatment			NICE	EAU	ICI	AUA
Female stress incontinence	Sub-urethral slings	TOT/TVT	Not recommended risk of urethral erosion.	LE III recommendation weak	LE: III; grade C	Moderate recommendation; Evidence level: grade C
		Minislings	N/A	N/A	N/A	N/A
		AFS	Procedure of choice	First-line treatment. Recommendation: strong	Preferable option Same evidence as synthetic slings	If considering an occlusive procedure
	AUS		Consider if other procedures are less likely to control incontinence.	Can be offered in experienced centers. Recommendation: weak.	Same as male but less experience	Less common than male but good success. Transvaginal cuff is a poor option.
	Bulking		N/A	See male	LE: III; grade C	Efficacy is modest and cure is rare. Conditional recommendation; evidence level: grade C
	Pro-ACT/ACT			See male	See male	Experimental, only in the context of clinical trials
Male stress incontinence	Slings	Advance	Same as female	Autologous and synthetic slings are an alternative		Experimental, only in clinical trials
		Atoms				
		Remeex				
		AFS				
	AUS		Same as Female	High success rate. LE III. Strong recommendation	Gold standard LE: III; grade C	Conditional recommendation; Evidence level: grade C
	Bulking		N/A	Positive results, early loss of continence		Low evidence
	Pro-ACT/ACT		N/A	Lower cure rate	Limited experience LE: III; grade C	See female
Bladder neck closure BNC				N/A	Mainly in children. LE: III; grade C	May be offered expert opinion
Anatomic/functional BOO	TURP			Consider sphincter function	In carefully selected patients	Careful selection
	Bladder neck resection			Only if sclerotic ring in cystoscopy.	LE: III; grade C	
	Urethrotomy			Same as no NLUTD	Tailored stepwise approach. LE: III; grade C	
	Sphincterotomy		N/A	Efficient for AD, irreversible need for reintervention	LE: III; grade C	High-risk of failure or need for additional surgery. Conditional Recommendation; Evidence level: grade C
	BNI			Not recommended if detrusor hypertrophy	Same as BN resection	
	Botox - Sphincter			Temporary solution. Not licensed.	Unclear: optimal dose and mode of injection. Duration about 3 months LE II; grade B	Not recommended for routine management
	Urethral stents			Comparable with sphincterotomy Costs, complications, and reinterventions are limiting factors.	Rarely indicated LE III; grade C	
	Balloon dilatation			No reports since 1994 No longer recommended.		
Denervation, deafferentation, neuromodulation	SARS			Charcot spinal arthropathy— long-term complication	LE: III; grade C	Promising outcomes Limited to investigational settings or specialty centers
	Sacral rhizotomy			Adjuvant to SARS		
	PTNS			TTNS recommended	Recommends TTNS and PTNS; grade of recommendation C.	Approved for non-neurogenic OAB Conditional recommendation for NLUTD; evidence level: grade C.
	Sacral neuromodulation		May be used in patients if conservative treatments unsuccessful	Growing evidence, unclear which neurological patients are most suitable.	LE II; grade B	Clinicians may offer to select NLUTD patients. Evidence level: grade C.

ACT, Adjustable continence therapy; AD, autonomic dysreflexia; AFS, Autologous fascial Sling; AUA, American Urological Association; AUS, Artificial Urinary Sphincter; BNI, bladder neck incision; EAU, European Association of Urology; ICI, International Consultation of Incontinence; NDO, neurogenic detrusor overactivity; NICE, National Institute for Health and Care Excellence; NLUTD, neurogenic lower urinary tract dysfunction; PTNS, percutaneous tibial nerve evaluation; SARS, sacral anterior root stimulation; TOT, Trans obturator tape; TURP, transurethral resection of the prostate; TVT, Tension-free vaginal tape.
